# Body mass index influences age-related cataracts: an updated meta-analysis and systemic review

**DOI:** 10.5935/0004-2749.2021-0382

**Published:** 2024-03-27

**Authors:** Huilin Chen, Xiaolei Sun, Li Pei, Ting Wang

**Affiliations:** 1 Department of Medical College, Qingdao University, Qingdao, China; 2 Eye Hospital of Shandong First Medical University, State Key Laboratory Cultivation Base, Shandong Provincial Key Laboratory of Ophthalmology, Shandong Eye Institute, Shandong First University & Shandong Academy of Medical Sciences, Jinan, China; 3 Department of Ocular Fundus, Eye Hospital of Shandong First Medical University, State Key Laboratory Cultivation Base, Shandong Provincial Key Laboratory of Ophthalmology, Shandong Eye Institute, Shandong First University & Shandong Academy of Medical Sciences, Jinan, China; 4 Department of Cataract, Eye Hospital of Shandong First Medical University, State Key Laboratory Cultivation Base, Shandong Provincial Key Laboratory of Ophthalmology, Shandong Eye Institute, Shandong First University & Shandong Academy of Medical Sciences, Jinan, China

**Keywords:** Aging, Cataracts, Body mass index, Overweight, Body weight, Obesity, Envelhecimento, Catarata, Índice de massa corporal, Sobrepeso, Obesidade

## Abstract

**Purpose:**

Visual impairment and blindness caused by cataracts are major public health problems. Several factors are associated with an increased risk of age-related cataracts, such as age, smoking, alcohol consumption, and ultraviolet radiation. This meta-analysis aimed to assess the association between body mass index and age-related cataracts.

**Methods:**

Studies on weight and age-related cataracts published from January 2011 to July 2020 were reviewed by searching PubMed, Medline, and Web of Science databases. The random-effects and fixed-effects models were used for the meta-analysis, and the results were reported as odd ratios.

**Results:**

A total of nine studies were included in the meta-analysis. No correlation was found between underweight and nuclear cataracts (OR=1.31, 95% CI [-0.50 to 3.12], p=0.156). The results of the random-effects model showed that overweight was significantly associated with age-related cataracts and reduced the risk of age-related cataracts (OR=0.91, 95% CI [0.80-1.02], p<0.0001; I^^2^^=62.3%, p<0.0001). Significant correlations were found between overweight and cortical, nuclear, and posterior subcapsular cataracts (OR=0.95, 95% CI [0.66-1.24], p<0.0001; OR=0.92, 95% CI (0.76-1.08), p<0.0001; OR=0.87, 95% CI [0.38-1.02], p<0.0001). Significant correlations were found between obesity and cortical, nuclear, and posterior subcapsular cataracts (OR=1.00, 95% CI [0.82-1.17], p<0.0001; OR=1.07, 95% CI [0.92-1.22], p<0.0001; OR=1.14, 95% CI [0.91-1.37], p<0.0001).

**Conclusion:**

This finding suggested a significant correlation between body mass index and age-related cataracts, with overweight and obesity reducing or increasing the risk of age-related cataracts, respectively.

## Abstract (pt)

**Objetivo:**

A deficiência visual e a cegueira causadas pela catarata são um grande problema de saúde pública. Há vários fatores associados a um risco maior de catarata relacionada à idade na população mundial, tais como idade, tabagismo, consumo de álcool e radiação ultravioleta. Esta meta-análise foi realizada para avaliar a associação entre o índice de massa corporal e a catarata relacionada à idade.

**Métodos:**

Foi revisada a literatura sobre catarata relacionada a peso e idade publicada de janeiro de 2011 a julho de 2020, através de buscas nos bancos de dados *PubMed, Medline* e *Web of Science*. Na meta-análise, foram utilizados modelos de efeito aleatórios e de efeitos fixos e os resultados foram apresentados como razões de chances (OR).

**Resultados:**

Um total de 9 estudos foi incluído na meta-análise. Não houve correlação entre ausência de sobrepeso e cataratas nucleares (OR=1,31, IC 95%: -0,50-3,12, p=0,156). Os resultados do modelo de efeitos aleatórios mostraram que o excesso de peso estava significativamente associado a uma redução do risco de catarata relacionada à idade (OR=0,91, IC 95%: 0,80-1,02, p<0,0001, I^^2^^=62,3%, p<0,0001). Houve correlações significativas entre o excesso de peso e cataratas corticais (OR=0,95, IC 95%: 0,66-1,24, p<0,0001), nucleares (OR=0,92, IC 95%: 0,76-1,08, p<0,0001) e subcapsulares posteriores (OR=0,87, IC 95%: 0,38-1,02, p<0,0001) relacionadas à idade. Houve correlações significativas entre obesidade e cataratas corticais (OR=1,00, IC 95%: 0,82-1,17, p<0,0001), nucleares (OR=1,07, IC 95%: 0,92-1,22, p<0,0001) e subcapsulares posteriores (OR=1,14, IC 95%: 0,91-1,37, p<0,0001) relacionadas à idade.

**Conclusão:**

Estes achados sugeriram uma correlação significativa entre o índice de massa corporal e a catarata relacionada à idade, com o excesso de peso e a obesidade reduzindo e aumentando o risco de catarata relacionada à idade, respectivamente.

## INTRODUCTION

Age-related cataracts (ARC) are a common ophthalmic disease, with common symptoms such as lens opacity and visual impairment^(1^,^2)^. Opacities originate in the nucleus, cortex, or posterior pole of the lens, resulting in nuclear cataracts (Nc), cortical cataracts (Cc), or posterior subcapsular cataracts (PSC), respectively^(3)^. Although the pathogenesis of ARC has not been fully elucidated, several studies have identified several factors that may increase the risk of ARC such as age, smoking, alcohol consumption, and obesity^(4^-^6)^. A recent study showed that diabetes was associated with incident Cc, and hypertension was associated with PSC cataract incidence^(7)^.

The World Health Organization defines normal body mass index (BMI) as 18.5-25 kg/m^2^, overweight as 25-30 kg/m^2^, and obese as >30 kg/m^^2^(8)^. Interestingly, Noran et al. did not find an association between BMI and ARC^(9)^. Conversely, Tan et al. and Mares et al. have reported that BMI was associated with an increased risk of ARC^(10^,^11)^. A study reported that the high BMI group showed a lower risk of developing cataracts than the normal BMI group^(12)^. Furthermore, some studies have reported that a high BMI and a low BMI might be associated with an increased risk of developing cataracts^(6^,^10^-^14)^. Three different types of cataracts affect different parts of the lens; however, inconsistencies in BMI-associated types of cataracts have been reported, i.e., a higher BMI was positively associated with Cc but negatively associated with Nc^(14)^. Two meta-analyses found an overall BMI/obesity association with cataracts, especially PSC^(15^,^16)^. The relationship between BMI and ARC risk is still controversial. Therefore, we conducted a systematic review and meta-analysis to quantitatively summarize the results regarding the effect of BMI on different ARC risk types.

## METHODS

This meta-analysis was performed following the PRISMA guidelines^(17)^.

### Search strategy

Studies published in English for the correlation between BMI and ARC were systematically searched from the databases of PubMed, Medline, and Web of Science from January 2011 to July 2020. The key words were as follows: (“body mass index” OR “obesity” OR “obese” OR “overweight” OR “body weight” OR “BMI”) AND (“cataract” OR “age-related cataract” OR “ARC”). The title, abstract, and complete manuscript were read to understand these studies. The reference lists of relevant meta-analyses were screened to identify studies that might have been missed.

### Inclusion and exclusion criteria

The inclusion criteria were as follows: (1) observational studies; (2) the outcome measures were any cataract, Cc, Nc, and PSC; (3) BMI categories were provided; (4) the correlation between BMI and ARC was investigated; and (5) relative risk (RR), odd ratios (OR), and 95% confidence intervals (CI) for BMI categories related the risk of cataracts were presented.

The exclusion criteria were as follows: (1) studies published as letters, conference papers and abstracts, animal studies, notes, meta-analysis, and systematic reviews, (2) no relevant literature information, and (3) repeated reports and reviews.

### Study selection

Two professionally trained researchers independently screened the studies and extracted the necessary information. In the case of any disagreement, a third party who has received professional training was involved in the discussion. Duplicate studies were expurgated using the delete option of the software Endnote X9. On the basis of the criteria, relevant studies with full texts available were identified. Then, the titles and abstracts were read to exclude studies whose study participants and type did not match the criteria. The contents of the studies that fulfill the inclusion criteria were further read, excluding those that were repeatedly published, with incomplete data and poor credibility.

### Data extraction

Data from the included studies were independently drawn by two investigators. Extraction differences between the two researchers we resolved by discussion with third parties. The indices extracted by the two researchers included author names, year of publication, sample size, age, BMI category, disease category, and control of confounding. The World Health Organization defines BMI <18.5 kg/m^2^ as underweight, 18.5-25 kg/m^2^ as normal, 25-30 kg/m^2^ as overweight, and 30 kg/m^2^ as obese^(8)^. BMI was used as an independent variable in this study, and any subtype of ARC was used as an outcome indicator.

### Assessment of study quality

The quality of the included studies was assessed using the Agency for Healthcare Research and Quality (AHRQ). The AHRQ scale has 11 items. If the answer is “no” or “unclear”, then the score is “0”. If the answer is “yes”, then the study scores “1”. The study was then assigned to the following categories corresponding to low quality (0-3), medium quality (4-7), and high quality (8-11).

### Statistical analysis

Data analysis was conducted using STATA14.0. The odds ratio (OR) and 95% confidence interval (CI) for the association between BMI and ARC were calculated to express the pooled effects. Random-effects or fixed-effects models were selected according to the heterogeneity of the test results. The Q test and I^2^ test were used to estimate the inter-study heterogeneity. When p>0.1 and I^2^≤50%, the fixed-effects model was used. When p<0.1 and I^2^≥50%, the random-effects model was adopted. Funnel plot and Egger tests were drawn to test whether there was a deviation in the included studies. P<0.05 was considered statistically significant.

## RESULTS

### Literature search and characteristics of the included studies

**Figure 1:**
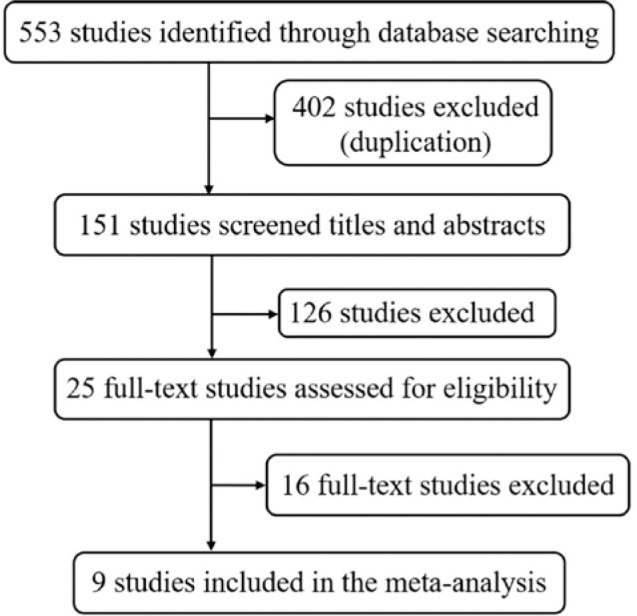
Flow diagram of the study selection process.

A total of 553 studies were retrieved from the database according to the search terms. After deleting duplicates (n=402) using Endnote X9, 151 remained. After reading titles and abstracts, 126 irrelevant references were excluded, and 25 full-text studies were assessed for eligibility. Based on the specified inclusion and exclusion criteria, 16 studies were excluded, and the final nine studies were included. The specific retrieval process is shown in figure 1.

The basic information of the included studies is shown in table 1. All the study participants were over 40 years old, and seven studies controlled for age and other confounding factors. Three studies clarified the relationship between underweight and cataract type. Seven studies reported an association between overweight and cataract types. Eight studies analyzed the relationship between the patient’s condition and the cataract type. Three studies analyzed the correlation between under-weight and Cc and Nc^(12^,^18^,^19)^. Seven studies focused on ARC and overweight^(7^,^12^,^18^-^22)^. Eight stduies examined ARC and obesity^(7^,^12^,^18^,^19^,^21^-^24)^. According to the quality evaluation of the included studies, six were of moderate quality and three were of high quality (Table 1).

**Table 1:** Characteristics of studies included in the meta-analysis

Authors (year)	Sample size	Age	BMI categories	Type of cataract	Confounding controlled	AHRQ
Sabanayagam et al. (2011)	2794	40-80	Overweight, obese	Any type	Adjusted for age, sex, education, and smoking status	8
Zhi-Quan Lu et al. (2012)	362	45-85	Underweight overweight, and obese	Any type	Adjusted for age and multiple potential confounders	8
Sangshin Park et al. _Men (2013)	1421	≥50	Underweight overweight, and obese	Any type, Cc, and Nc	Adjusted for age, vigorous physical activity, smoking, alcohol consumption, sunlight exposure, diabetes mellitus, and education level, income	5
Sangshin Park et al. _ Women (2013)	1827	≥50				
Sangshin Park et al. _Men (2014)	1191	55.7	Obese	Any type, Cc, and Nc	Adjusted for age, daily time spent in vigorous physical activity, total pack-years of smoking, amount of daily alcohol consumption, daily sun exposure, family income, occupational status, marital status, and family history of eye disease	4
Sangshin Park et al. _Women (2015)	1661	61.4				
P K Nirmalan et al. (2015)	2499	≥40	Overweight and obese	Any type, Cc, Nc, and PSC	Adjusted for age	4
Deok-Soon Lee et al. _ Men (2015)	2134	50.3	Overweight	Any type, Cc, and Nc	Adjusted for age, income, occupation, smoking, drinking, education, sun exposure, diabetes, and high cholesterol, and hypertension	6
Deok-Soon Lee et al. _Women (2015)	2780	50.1				
Sumeer Singh et al. _rural (2019)	1094	66.4	Underweight overweight, and obese	Any type	Not mentioned	7
Sumeer Singh et al. _ urban (2019)	649	67.8				
Ava Grace Tan et al. (2019)	1094	66.4	Obese	Cc, Nc, and PSC	Adjusted for age, sex, smoking status, hypertension, diabetes, education, myopia, steroid use	8
Ava Grace Tan et al. (2020)	3580	≥49	Overweight and obese	Cc, Nc, and PSC	Adjusted for age, sex, current smoking, hypertension, diabetes, socioeconomic status, BCVA, myopia, CKD, CRP, CVD, and total cholesterol	7

AHRQ= Agency for Healthcare Research and Quality; any type, any type cataracts; Cc= cortical cataracts; Nc= nuclear cataracts; PSC= posterior subcapsular cataracts.

### Association between underweight and ARC

**Figure 2:**
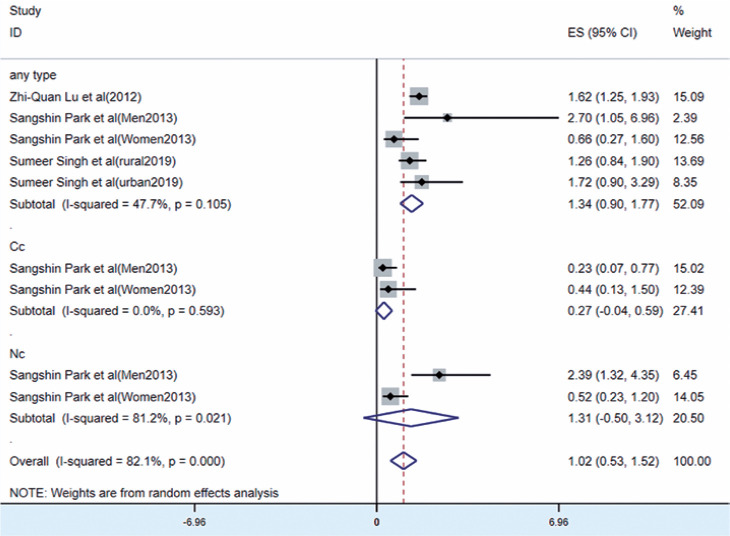
Forest plot of risk estimates of ARC associated with underweight.

Three studies^(12^,^18^,^19)^ reported the relationship between underweight and ARC, as shown in figure 2. The results of the random-effects model suggested that being underweight was a risk factor for ARC (OR=1.02, 95% CI [0.53, 1.52], p<0.0001; I^2^=81.2%, p<0.0001). No sig-nificant correlation was found between underweight and Cc (OR=0.27, 95% CI [-0.04, 0.59], p=0.085). Underweight was not correlated with Nc (OR=1.31, 95% CI [-0.50, 3.12], p=0.156).

CI= confidence interval; ES= effect size; ID= identification.

### Association between overweight and ARC

**Figure 3:**
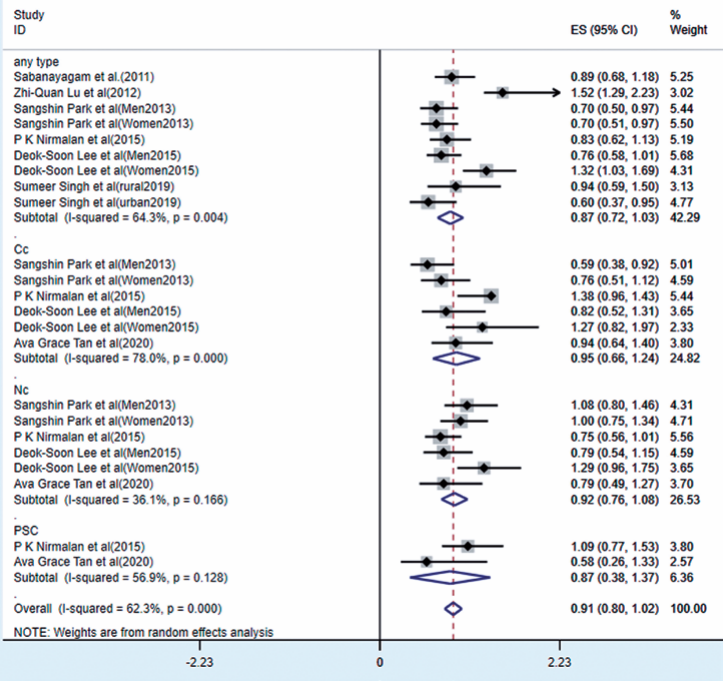
Forest plot of risk estimates of ARC associated with overweight.

**Figure 4:**
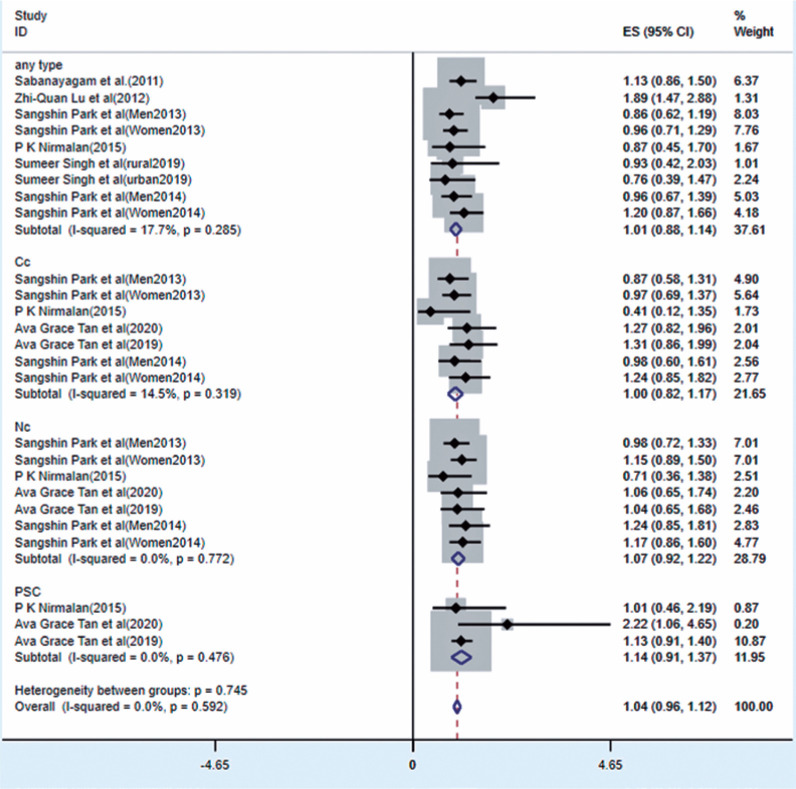
Analysis of publication bias about overweight using funnel plot.

As shown in figure 3, seven studies reported the relationship between overweight and ARC. The results of the random-effects model showed that overweight was significantly associated with ARC and reduced the risk of ARC (OR=0.94, 95% CI [0.85-1.03], p<0.0001; I^2^=62.3%, p<0.0001). Significant correlations were found between overweight and Cc (OR=0.95, 95% CI [0.66-1.24], p<0.0001), Nc (OR=0.92, 95% CI [0.76-1.08], p<0.0001), and PSC (OR=0.87, 95% CI [0.38-1.02], p<0.0001), respectively. According to the funnel plot and Egger test results, no bias was found among the studies (p=0.971, Figure 4).

CI= confidence interval; ES= effect size; ID= identification.

any type= any cataract; Cc= cortical cataracts; Nc= nuclear cataracts; PSC= posterior subcapsular cataracts; CI= confidence interval.

### Association between obesity and ARC

**Figure 5:**
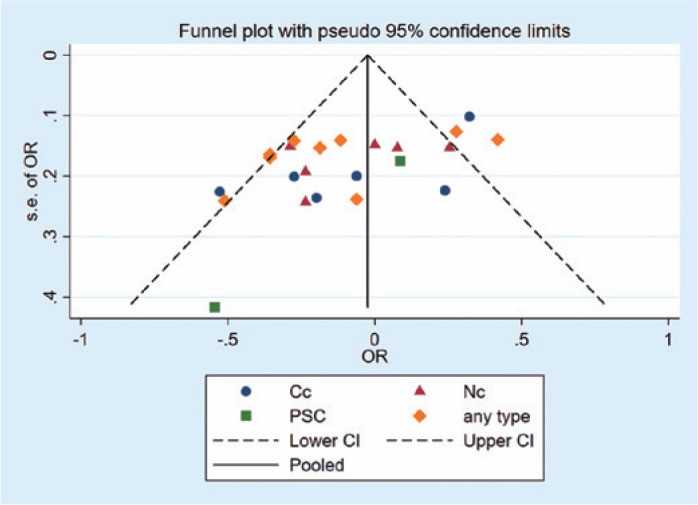
Forest plot of risk estimates of ARC associated with obesity.

**Figure 6:**
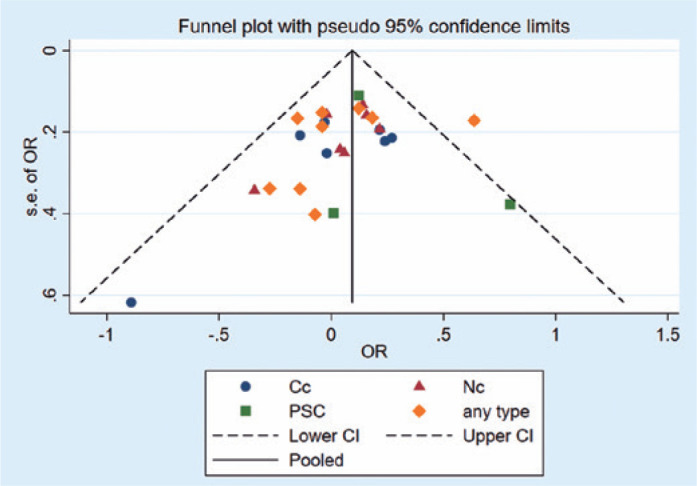
Analysis of publication bias about obesity using funnel plot.

Eight studies reported a relationship between obesity and ARC (Figure 5). The results of the fixed-effects model showed that obesity was significantly associated with ARC, increasing the risk of cataracts (OR=1.05, 95% CI [0.98-1.13], p<0.0001; I^^2^^=0.0%, p=0.592). Significant correlations were found between obesity and Cc (OR=1.00, 95% CI [0.82-1.17], p<0.0001), Nc (OR=1.07, 95% CI [0.92-1.22], p<0.0001), and PSC (OR=1.14, 95% CI [0.91-1.37], p<0.0001), respectively. According to the funnel plot and Egger’s test, no bias was found among the studies (p=0.948, Figure 6).

CI= confidence interval; ES= effect size; ID= identification.

Cc= cortical cataracts; Nc, nuclear cataracts; PSC= posterior subcapsular cataracts; any type= any cataract; CI= confidence interval.

## DISCUSSION

BMI is one of the few potentially modifiable risk factors of cataract formation, although the exact mechanism for cataract formation is unclear. A healthy lifestyle can maintain the BMI in the normal range. This can bring benefits to health and help reduce the incidence of cataracts and associated costs. The relationship between BMI and cataract risk is still controversial in observational studies. BMI was reported to be positively correlated with cataract risk, the risk of heavier weight was lower, but no significant relationship was found^(25)^. Different types of cataracts can result from different causes^(26^,^27)^. Clinically, whether underweight, overweight, and obesity are associated with a particular type of cataracts should be determined because the pathophysiology, treatment, and effect of these three subtypes (Nc, Cc, and PSC) on visual function tend to be different. Therefore, a systematic approach to combine the results of all available studies evaluating the longitudinal association of underweight, overweight, and obesity with ARC subtypes would be beneficial.

This meta-analysis of nine studies on the classification relationship between BMI and ARC showed that being underweight was significantly associated with the risk of Nc, not with Cc. Interestingly, Park et al. reported that underweight (low BMI) negatively correlated with Cc (OR=0.22, 95% CI [0.07-0.73]), but positively correlated with Nc (OR=2.36, 95% CI [1.35-4.11])^(12)^. This study also indicated that being overweight was significantly associated with ARC risk. This conclusion is similar to published ones. For example, Kuang et al. reported that BMI significantly correlated with Nc and Cc and negatively to each other. As BMI increases from 21, the Nc risk decreases. When BMI approached^(28)^, this protective effect stopped, and the risk of NC increased as the BMI increased to the obese level. On the contrary, the Cc risk increased gra-dually as BMI increased from 21 to normal weight, whereas the risk of Cc decreased when BMI was >28. BMI was not associated with PSC in this study^(14)^.

Furthermore, many studies have suggested possible pathological causes and mechanisms of the association between obesity and cataracts. Three major mechanisms by which cataract development leads to lens damage, including oxidative stress, osmotic action, and non-enzymatic protein glycosylation^(28)^, to any or all of which obesity may affect the physiological processes^(29^,^30)^. In a recent review article, Cheung and Wong reported that obesity was most consistently related to Cc and PSC cataracts^(31)^. More interestingly, Noran et al. reported that only patients who were obese were 2.4 times more likely to develop ARC, and no association was found between BMI and ARC^(9)^. Obesity was reported to be significantly correlated with Cc and PSC, but not with Nc^(5)^. However, in our study, the incidence of cataracts decreased in the overweight group, including Nc, Cc, and SPC. Therefore, BMI was not positively correlated with cataract risk. Similar to the findings of Park et al., the overweight group had a significantly lower risk of cataract development than the normal-weight group^(31)^. However, some studies had different conclusions. For example, studies have found that obesity and increased BMI significantly correlated with PSC or Nc^(11^,^32)^. Moreover, other studies have shown that higher BMI could predict the occurrence of PSC and Nc, but not Cc^(33^,^34)^. These differences may be attributed to genetic, demographic, or environmental differences or different methods in statistical analysis.

These findings suggested a significant correlation between BMI and ARC, with overweight and obesity reducing or increasing the risk of ARC, respectively. The research on the relationship between BMI and cataract development is still not clearly known and needs to be explored. In conclusion, BMI obtained from a simple physical examination is useful in predicting cataracts. However, this study has some limitations. First, overweight and obesity are risk factors for various diseases and may co-exist with diabetes, hypertension, coronary heart disease, gout, and depression^(35^-^38)^, which may mediate the association between BMI and cataracts^(39^-^43)^. Second, some of the studies used different cataract grading systems, which would affect the estimation of BMI and cataracts. Finally, given the lack of participant and personnel blinding, most of the studies have a high risk of bias; thus, subjective impressions can affect the results. Publication bias exists in this study; however, the degree of bias was acceptable. In consideration of these limitations, randomized trials are needed to future examine the ARC risk with BMI reduction.
